# The disc separation and the eigenvalue distribution of the Schur complement of nonstrictly diagonally dominant matrices

**DOI:** 10.1186/s13660-017-1340-0

**Published:** 2017-04-05

**Authors:** Cheng-yi Zhang, Weiwei Wang, Shuanghua Luo, Jianxing Zhao

**Affiliations:** 1 School of Science, Xi’an Polytechnic University, Xi’an, Shaanxi 710048 China; 2 College of Science, Guizhou Minzu University, Guiyang, Guizhou 550025 China

**Keywords:** 15A06, 15A15, 15A48, Geršgorin disc separation, Schur complement, nonstrictly diagonally dominant matrices, eigenvalue distribution

## Abstract

The result on the Geršgorin disc separation from the origin for strictly diagonally dominant matrices and their Schur complements in (Liu and Zhang in SIAM J. Matrix Anal. Appl. 27(3):665-674, 2005) is extended to nonstrictly diagonally dominant matrices and their Schur complements, showing that under some conditions the separation of the Schur complement of a nonstrictly diagonally dominant matrix is greater than that of the original grand matrix. As an application, the eigenvalue distribution of the Schur complement is discussed for nonstrictly diagonally dominant matrices to derive some significant conclusions. Finally, some examples are provided to show the effectiveness of theoretical results.

## Introduction

Let $A=(a_{ij})\in\mathbb{C}^{n\times n}$ with $R_{i}(A)=\sum_{j=1,j\neq i}^{n} \vert a_{ij} \vert $ for all $i\in\langle n\rangle$ define the disc separation of *A* by the quantities $\vert a_{ii} \vert -R_{i}(A)$ that measure the separations of the discs

$$\vert z-a_{ii} \vert \leq R_{i}(A), \quad i=1,2,\ldots, n, $$

from the origin and give estimate $\min_{1\leq i\leq n} \vert a_{ii} \vert -R_{i}(A)$ of the absolute value of the shortest eigenvalue (the eigenvalue with the smallest absolute value, see [1]) of the strictly diagonally dominant matrix $A\in\mathbb {C}^{n\times n}$.

In 2005, Liu and Zhang [1] firstly studied the disc separation of the Schur complements of strictly diagonally dominant matrices. More precisely, they compared a disc separation of the Schur complement to that of the original matrix and showed that each Geršgorin disc of the Schur complement is paired with a particular Geršgorin disc of the original matrix; the latter is further from the origin than the former. Their result is as follows.

### Theorem 1

see [1]

*Given an* $n\times n$ *strictly diagonally dominant matrix* $A=(a_{ij})$ *and two sets* $\alpha=\{i_{1},i_{2},\ldots, i_{m}\}\subset\langle n\rangle=\{1,2,\ldots ,n\}$ *and* $\alpha'=\langle n\rangle-\alpha=\{j_{1},j_{2},\ldots, j_{l}\} \subset\langle n\rangle$ *with* $m+l=n$, *let*

1 $$ \omega_{j_{t}}=\min_{1\leq v\leq m} \frac{ \vert a_{i_{v}i_{v}} \vert -R_{i_{v}}}{ \vert a_{i_{v}i_{v}} \vert }\sum_{u=1}^{m} \vert a_{j_{t}i_{u}} \vert $$

*for all* $j_{t}\in\alpha'$, *and define* $A/\alpha=(\widetilde {a}_{j_{t},j_{s}})$, *then*

2 $$ \vert \widetilde{a}_{j_{t},j_{t}} \vert -R_{j_{t}}(A/\alpha )\geq \vert {a}_{j_{t},j_{t}} \vert -R_{j_{t}}(A)+\omega _{j_{t}}\geq \vert {a}_{j_{t},j_{t}} \vert -R_{j_{t}}(A)>0 $$

*and*

3 $$ \vert \widetilde{a}_{j_{t},j_{t}} \vert +R_{j_{t}}(A/\alpha )\leq \vert {a}_{j_{t},j_{t}} \vert +R_{j_{t}}(A)-\omega _{j_{t}}\leq \vert {a}_{j_{t},j_{t}} \vert +R_{j_{t}}(A). $$

On the other hand, Liu and Zhang [1] also improved the result of Theorem 1 in [2] when $A\in \mathit{SD}_{n}\subset H^{S}_{n}$ and established the new result on the eigenvalue distribution for the Schur complements of strictly diagonally dominant matrices with real diagonal entries.

### Theorem 2

see [1]

*Let* $A=(a_{ij})$ *be an* $n\times n$ *strictly diagonally dominant matrix with real diagonal entries*, *and* $\alpha\subset\langle n\rangle$. *Then* $A/\alpha$ *and* $A(\alpha')$ *have the same number of eigenvalues whose real parts are greater* (*less*) *than* *w* (*resp*. −*w*), *where* $\alpha'=\langle n\rangle-\alpha$ *and*

4 $$ w=\min_{j\in \alpha'} \biggl[ \vert a_{jj} \vert -R_{j}(A)+\min_{i\in \alpha} \frac{ \vert a_{ii} \vert -R_{i}(A)}{ \vert a_{ii} \vert }\sum_{i\in \alpha} \vert a_{ji} \vert \biggr]. $$

In this paper, we will generalize the result on the Geršgorin disc separation from the origin for strictly diagonally dominant matrices and their Schur complements in [1] to nonstrictly diagonally dominant matrices and their Schur complements, showing that under some conditions the separation of the Schur complement of a nonstrictly diagonally dominant matrix is greater than that of the original grand matrix. As an application, we continue discussing the eigenvalue distribution of the Schur complements for nonstrictly diagonally dominant matrices to derive some significant conclusions.

The paper is organized as follows. Some notations and preliminary results about nonstrictly diagonally dominant matrices are given in Section 2. Some results on the Geršgorin disc separation from the origin are established in Section 3 for nonstrictly diagonally dominant matrices and their Schur complements. The eigenvalue distribution of the Schur complements is discussed in Section 4 for nonstrictly diagonally dominant matrices to derive some significant conclusions. Some examples are provided in Section 5 to show the effectiveness of theoretical results. Conclusions are given in Section 6.

## Preliminaries

In this section we give some notions and preliminary results about special matrices that are used in this paper.

$\mathbb {C}^{m\times n} (\mathbb{R}^{m\times n})$ will be used to denote the set of all $m\times n$ complex (real) matrices.

$\mathbb{Z}$ denotes the set of all integers. Let $\alpha\subseteq \langle n\rangle=\{1,2,\ldots,n\}\subset\mathbb{Z}$. $\vert \alpha \vert $ denotes the cardinality of the set *α*. For nonempty index sets $\alpha,\beta\subseteq\langle n\rangle$, $A(\alpha,\beta)$ is the submatrix of $A\in \mathbb {C}^{n\times n}$ with row indices in *α* and column indices in *β*. The submatrix $A(\alpha,\alpha)$ is abbreviated to $A(\alpha)$. *Let* $A\in \mathbb {C}^{n\times n}$ *,* $\alpha\subset\langle n\rangle$ *and* $\alpha^{\prime}=\langle n\rangle-\alpha$ *. If* $A(\alpha)$ *is nonsingular, the matrix*

5 $$ A/\alpha=A\bigl(\alpha^{\prime}\bigr)-A\bigl( \alpha^{\prime},\alpha\bigr)\bigl[A(\alpha )\bigr]^{-1}A\bigl( \alpha,\alpha^{\prime}\bigr) $$

*is called the Schur complement with respect to* $A(\alpha)$ *, indices in both* *α* *and* $\alpha^{\prime}$ *are arranged with increasing order.* We shall confine ourselves to the nonsingular $A(\alpha)$ as far as $A/\alpha$ is concerned.

A matrix $A=(a_{ij})\in\mathbb{R}^{n\times n}$ is called *nonnegative* if $a_{ij}\geq0$ for all $i,j\in \langle n\rangle$. *A matrix* $A=(a_{ij})\in\mathbb{R}^{n\times n}$ *is called a* *Z-matrix if* $a_{ij}\leq0$ *for all* $i\neq j$ *.* We will use $Z_{n}$ to denote the set of all $n\times n$ *Z*-matrices. *A matrix* $A=(a_{ij})\in Z_{n}$ *is called an* *M-matrix if* *A* *can be expressed in the form* $A=sI-B$ *, where* $B\geq0$ *, and* $s\geq \rho(B)$ *, the spectral radius of* *B. If* $s>\rho(B)$ *,* *A* *is called a nonsingular* *M-matrix* $M_{n}$ and $M^{\bullet}_{n}$ will be used to denote the set of all $n\times n$ *M*-matrices and the set of all $n\times n$ nonsingular *M*-matrices, respectively (see [3]).

*The comparison matrix of a given matrix* $A=(a_{ij})\in \mathbb {C}^{n\times n}$ *, denoted by* $\mu(A)=(\mu_{ij})$ *, is defined by*

6 $$ \mu_{ij}= \textstyle\begin{cases} \vert a_{ii} \vert , & \text{if } i=j,\\ - \vert a_{ij} \vert , & \text{if } i\neq j. \end{cases} $$

It is clear that $\mu(A)\in Z_{n}$ for a matrix $A\in \mathbb{C}^{n\times n}$. *A matrix* $A=(a_{ij})\in\mathbb {C}^{n\times n}$ *is called a general* *H-matrix if* $\mu(A)\in M_{n}$ *. If* $\mu(A)\in M^{\bullet}_{n}$ *,* *A* *is called an invertible* *H-matrix.* $H_{n}$ and $H^{I}_{n}$ will denote the set of all $n\times n$ general *H*-matrices and the set of all $n\times n$ invertible *H*-matrices, respectively (see [4]).

### Lemma 1

see [2]

*If* $A\in H^{I}_{n}$, *then*

7 $$ \bigl[\mu(A)\bigr]^{-1}\geq \bigl\vert A^{-1} \bigr\vert \geq0. $$

*For* $n\geq2$, *an* $n\times n$ *complex matrix* *A* *is reducible if there exists an* $n\times n$ *permutation matrix* *P* *such that*

8 $$ PAP^{T} = \left [ \textstyle\begin{array}{c@{\quad}c} A_{11} & A_{12}\\ 0 & A_{22} \end{array}\displaystyle \right ], $$

*where* $A_{11}$ *is an* $r\times r$ *submatrix and* $A_{22}$ *is an* $(n-r)\times(n-r)$ *submatrix*, *where* $1 \leq r< n$. *If no such permutation matrix exists*, *then* *A* *is called irreducible*. *If* *A* *is a* $1\times1$ *complex matrix*, *then* *A* *is irreducible if its single entry is nonzero*, *and reducible otherwise*.

A matrix $A\in \mathbb {C}^{n\times n}$ is called diagonally dominant by row if

9 $$ \vert a_{ii} \vert \geq\sum _{j=1,j\neq i}^{n} \vert a_{ij} \vert $$

holds for all $i\in\langle n\rangle$. If inequality in (9) holds strictly for all $i\in\langle n\rangle$, *A* is called strictly diagonally dominant by row. If *A* is irreducible and the inequality in (9) holds strictly for at least one $i\in \langle n\rangle$, *A* is called irreducibly diagonally dominant by row. If (9) holds with equality for all $i\in\langle n\rangle$, *A* is called diagonally equipotent by row. If (9) holds with equality for at least one $i\in \langle n\rangle$, *A* is called nonstrictly diagonally dominant.

$D_{n}(\mathit{SD}_{n}, \mathit{ID}_{n})$ and $\mathit{DE}_{n}$ will be used to denote the sets of all $n\times n$ (strictly, irreducibly) diagonally dominant matrices and the set of all $n\times n$ diagonally equipotent matrices, respectively.

### Lemma 2

see [5]

*Let* $A\in D_{n}$. *Then* *A* *is singular if and only if there exists a singular principal submatrix in A*.

### Lemma 3

see [5]

*Let* $A\in D_{n}$. *Then* $A\in H^{I}_{n}$ *if and only if* *A* *has either one zero principal submatrix or one irreducibly diagonally equipotent principal submatrix*. *Furthermore*, *if* $A\in D_{n}\cap Z_{n}$, *then* $A\in M^{\bullet}_{n}$ *if and only if* *A* *has either one zero principal submatrix or one irreducibly diagonally equipotent principal submatrix*.

### Lemma 4

see Lemma 3.4 in [6]

*Given a matrix* $A\in D_{n}$ *and a set* $\alpha=\langle n\rangle-\alpha'\subseteq\langle n\rangle$, *if* $A(\gamma)$ *is the largest diagonally equipotent principal submatrix of* $A(\alpha)$ *for* $\gamma=\alpha-\gamma'\subseteq\alpha$, *then* ${A/\alpha}=A(\alpha'\cup\gamma')/\gamma'$, *where*

10 $$ A\bigl(\alpha^{\prime}\cup\gamma'\bigr) = \left [ \textstyle\begin{array}{c@{\quad }c} A(\gamma') & A(\gamma',\alpha^{\prime})\\ A(\alpha^{\prime},\gamma') & A(\alpha^{\prime}) \end{array}\displaystyle \right ]. $$

### Lemma 5

see [1]

*Let* $A \in C^{n\times n}$ *be partitioned as*

$$A=\left ( \textstyle\begin{array}{c@{\quad}c} a_{11} & A_{12}\\ A_{21} & A_{22} \end{array}\displaystyle \right ), $$

*where* $A_{21}=(a_{21},a_{31},\ldots,a_{n1})^{T}$ *and* $A_{12}=(a_{12},a_{13},\ldots,a_{1n})$. *If* $A_{22}$ *is nonsingular*, *then*

$$\begin{aligned}& \frac{\det A}{\det A_{22}}=a_{11}-(a_{12},a_{13}, \ldots,a_{1n})[A_{22}]^{-1} \left ( \textstyle\begin{array}{c} a_{21}\\ \vdots\\ a_{n1} \end{array}\displaystyle \right ). \end{aligned}$$

## The disc separation of the Schur complement of nonstrictly diagonally dominant matrices

In this section, we will establish some results on the Geršgorin disc separation from the origin for nonstrictly diagonally dominant matrices and their Schur complements such that under some conditions the separation of the Schur complement of a nonstrictly diagonally dominant matrix is greater than that of the original grand matrix. Firstly, the following lemma will be used in the rest of this subsection.

### Lemma 6

*Let* $A=(a_{ij})\in D_{n}$ *be nonsingular*, *and two sets* $\alpha=\{i_{1},i_{2},\ldots, i_{m}\}\subset\langle n\rangle$ *and* $\alpha '=\langle n\rangle-\alpha=\{j_{1},j_{2},\ldots, j_{l}\}\subset\langle n\rangle$ *with* $m+l=n$. *For any* $j_{t}\in\alpha'$, *denote*

11 $$ B_{jt}=\left [ \textstyle\begin{array}{c@{\quad}c@{\quad}c@{\quad}c} x & - \vert a_{j_{t}i_{1}} \vert & \cdots & - \vert a_{j_{t}i_{m}} \vert \\ -\sum_{i=1}^{l} \vert a_{i_{1},j_{i}} \vert & & & \\ \vdots & & \mu[A(\alpha)] & \\ \vert a_{i_{m},j_{i}} \vert & & & \end{array}\displaystyle \right ]. $$

*Then* $B_{jt}$ *is doubly diagonally dominant* [7] *if and only if*

12 $$ x\geq\max_{1\leq\omega\leq m}\frac{R_{i_{\omega }}(A)}{ \vert a_{i_{\omega}i_{\omega}} \vert }. $$

*When inequality* (12) *holds*, $B_{jt}$ *is an* *M*-*matrix of order* $m+1$, *and thus*, $\det B_{jt}\geq0$. *Furthermore*, *if the strict inequality* (12) *holds and* $\vert a_{i_{s}i_{s}} \vert >R_{i_{s}}(A)$ *for all* $i_{s}\in\alpha$, *then* $B_{jt}$ *is a nonsingular* *M*-*matrix of order* $m+1$, *and thus*, $\det B_{jt}>0$.

### Proof

Similar to the proof of Lemma 4 in [1], we can easily derive the conclusion of this lemma. □

### Theorem 3

*Let* $A=(a_{ij})\in D_{n}$ *be nonsingular*, *and two sets* $\alpha=\{i_{1},i_{2},\ldots, i_{m}\}\subset\langle n\rangle$ *and* $\alpha '=\langle n\rangle-\alpha=\{j_{1},j_{2},\ldots, j_{l}\}\subset\langle n\rangle$ *with* $m+l=n$, *define* $A/\alpha=(\widetilde{a}_{j_{t},j_{s}})$ *and* $\omega_{j_{t}}$ *as in* (1), *if*

13 $$ \vert {a}_{j_{t},j_{t}} \vert >R_{j_{t}}(A) $$

*for all* $j_{t}\in\alpha'$, *then both* (2) *and* (3) *hold*.

### Proof

Since $A\in D_{n}$ and is nonsingular, Lemma 2 indicates that ${A(\alpha)}$ is nonsingular. As a result, $A/\alpha =(\widetilde{a}_{j_{t},j_{s}})$ exists. According to definition (5) of the Schur complement matrix $A/\alpha$, we have the off-diagonal entries

14 $$ \begin{aligned}[b] &\widetilde{a}_{j_{l},j_{t}}=a_{j_{l},j_{t}}- \left [(a_{j_{l},i_{1}},a_{j_{l},i_{2}},\ldots,a_{j_{l},i_{k}})\bigl[A( \alpha)\bigr]^{-1} \left ( \textstyle\begin{array}{c} a_{i_{1},j_{t}}\\ a_{i_{2},j_{t}}\\ \vdots\\ a_{i_{k},j_{t}} \end{array}\displaystyle \right ) \right ], \\ & \quad l,t=1,2,\ldots,m, \end{aligned} $$

and the diagonal entries

15 $$ \begin{aligned}[b] &\widetilde{a}_{j_{l},j_{l}}=a_{j_{l},j_{l}}- \left [(a_{j_{l},i_{1}},a_{j_{l},i_{2}},\ldots,a_{j_{l},i_{k}})\bigl[A( \alpha)\bigr]^{-1} \left ( \textstyle\begin{array}{c} a_{i_{1},j_{l}}\\ a_{i_{2},j_{l}}\\ \vdots\\ a_{i_{k},j_{l}} \end{array}\displaystyle \right ) \right ], \\ &\quad l=1,2,\ldots,m, \end{aligned} $$

of $A/\alpha$. The conclusion of this theorem will be proved by proving the following two cases:

(i) If $A(\alpha)\in H^{I}_{ \vert \alpha \vert }$, Lemma 1 gives

16 $$ \bigl\{ \mu\bigl[A(\alpha)\bigr]\bigr\} ^{-1}\geq \bigl\vert \bigl[A(\alpha)\bigr]^{-1} \bigr\vert \geq0. $$

Then from (14), (15), (16) and Lemma 5, we have

17 $$\begin{aligned} \vert \widetilde{a}_{j_{t},j_{t}} \vert -R_{j_{t}}(A/ \alpha ) &= \vert \widetilde{a}_{j_{t},j_{t}} \vert -\sum _{i=1,i\neq t}^{l} \vert \widetilde{a}_{j_{t},j_{i}} \vert \\ & = \left \vert \left [a_{j_{t},j_{t}}- (a_{j_{t},i_{1}}, \ldots,a_{j_{t},i_{k}})\bigl[A(\alpha)\bigr]^{-1} \left ( \textstyle\begin{array}{c} a_{i_{1},j_{t}}\\ a_{i_{2},j_{t}}\\ \vdots\\ a_{i_{k},j_{t}} \end{array}\displaystyle \right ) \right ] \right \vert \end{aligned}$$

18 $$\begin{aligned} &\quad{} -\sum_{i=1,i\neq t}^{l} \left \vert \left [a_{j_{l},j_{i}}- (a_{j_{l},i_{1}},\ldots,a_{j_{l},i_{m}}) \bigl[A(\alpha)\bigr]^{-1} \left ( \textstyle\begin{array}{c} a_{i_{1},j_{i}}\\ a_{i_{2},j_{i}}\\ \vdots\\ a_{i_{k},j_{i}} \end{array}\displaystyle \right ) \right ] \right \vert \\ &\geq \vert a_{j_{l},j_{l}} \vert -\sum _{i=1,i\neq t}^{l} \vert a_{j_{l},j_{i}} \vert \\ &\quad{} -\sum_{i=1}^{l} \left \vert \left [ (a_{j_{t},i_{1}},a_{j_{t},i_{2}},\ldots,a_{j_{t},i_{m}}) \bigl[A(\alpha)\bigr]^{-1} \left ( \textstyle\begin{array}{c} a_{i_{1},j_{i}}\\ a_{i_{2},j_{i}}\\ \vdots\\ a_{i_{m},j_{i}} \end{array}\displaystyle \right ) \right ] \right \vert \\ &\geq \vert a_{j_{t},j_{t}} \vert -\sum _{i=1,i\neq t}^{l} \vert a_{j_{t},j_{i}} \vert \\ & \quad{} -\sum_{i=1}^{l}\left [ \bigl( \vert a_{j_{t},i_{1}} \vert , \vert a_{j_{t},i_{2}} \vert ,\ldots, \vert a_{j_{t},i_{m}} \vert \bigr) \bigl\vert \bigl[A(\alpha) \bigr]^{-1} \bigr\vert \left ( \textstyle\begin{array}{c} \vert a_{i_{1},j_{i}} \vert \\ \vert a_{i_{2},j_{i}} \vert \\ \vdots\\ \vert a_{i_{m},j_{i}} \vert \end{array}\displaystyle \right ) \right ] \\ &\geq \vert a_{j_{t},j_{t}} \vert -\sum _{i=1,i\neq t}^{l} \vert a_{j_{t},j_{i}} \vert \\ &\quad{} -\sum_{i=1}^{l}\left [ \bigl( \vert a_{j_{t},i_{1}} \vert , \vert a_{j_{t},i_{2}} \vert , \ldots, \vert a_{j_{t},i_{m}} \vert \bigr)\bigl\{ \mu\bigl[A(\alpha)\bigr] \bigr\} ^{-1} \left ( \textstyle\begin{array}{c} \vert a_{i_{1},j_{i}} \vert \\ \vert a_{i_{2},j_{i}} \vert \\ \vdots\\ \vert a_{i_{m},j_{i}} \vert \end{array}\displaystyle \right ) \right ] \\ &= \vert a_{j_{t},j_{t}} \vert -R_{j_{t}}(A)+\sum _{u=1}^{l} \vert a_{j_{t}i_{u}} \vert + \omega_{j_{t}}-\omega _{j_{t}} \\ &\quad{} -\sum_{i=1}^{l}\left [ \bigl( \vert a_{j_{t},i_{1}} \vert , \vert a_{j_{t},i_{2}} \vert ,\ldots, \vert a_{j_{t},i_{m}} \vert \bigr)\bigl\{ \mu\bigl[A(\alpha)\bigr] \bigr\} ^{-1} \left ( \textstyle\begin{array}{c} \vert a_{i_{1},j_{i}} \vert \\ \vert a_{i_{2},j_{i}} \vert \\ \vdots\\ \vert a_{i_{m},j_{i}} \vert \end{array}\displaystyle \right ) \right ] \\ &= \vert a_{j_{t},j_{t}} \vert -R_{j_{t}}(A)+ \omega_{j_{t}} +\sum_{u=1}^{m} \vert a_{j_{t}i_{u}} \vert -\omega _{j_{t}} \\ &\quad{} -\sum_{i=1}^{l}\left [ \bigl( \vert a_{j_{t},i_{1}} \vert , \vert a_{j_{t},i_{2}} \vert ,\ldots, \vert a_{j_{t},i_{m}} \vert \bigr)\bigl\{ \mu\bigl[A(\alpha)\bigr] \bigr\} ^{-1} \left ( \textstyle\begin{array}{c} \vert a_{i_{1},j_{i}} \vert \\ \vert a_{i_{2},j_{i}} \vert \\ \vdots\\ \vert a_{i_{m},j_{i}} \vert \end{array}\displaystyle \right ) \right ] \\ &= \vert a_{j_{t},j_{t}} \vert -R_{j_{t}}(A)+ \omega_{j_{t}}+\frac {\det B_{j_{t}}}{\det\mu[A(\alpha)]}, \end{aligned}$$

where

$$\begin{aligned}& {B_{j_{t}}}= \left( \textstyle\begin{array}{c@{\quad }c} \sum_{u=1}^{m} \vert a_{j_{t}i_{u}} \vert -\omega _{j_{t}} & h^{T}\\ g & \mu[A(\alpha)] \end{array}\displaystyle \right)_{(m+1)\times(m+1) }, \\& g = \Biggl(-\sum_{i=1}^{l} \vert a_{i_{1},j_{i}} \vert ,\ldots,-\sum_{i=1}^{l} \vert a_{i_{m},j_{i}} \vert \Biggr)^{T}, \\& h = \bigl(- \vert a_{j_{l},i_{1}} \vert ,\ldots ,- \vert a_{j_{l},i_{m}} \vert \bigr)^{T}. \end{aligned}$$

It is clear that $B_{j_{t}}\in Z_{m+1}$. In Lemma 6, we set

19 $$ x=\sum_{u=1}^{m} \vert a_{j_{t}i_{u}} \vert -\omega _{j_{t}}\geq\max_{1\leq i_{\omega}\leq m} \frac{R_{i_{\omega }}(A)}{ \vert a_{i_{\omega}i_{\omega}} \vert }. $$

According to Lemma 6, $\det B_{j_{t}}=\det\mu(B_{j_{t}})\geq0$. Continuing (17),

20 $$ \begin{aligned}[b] \vert \widetilde{a}_{j_{t},j_{t}} \vert -R_{j_{t}}(A/\alpha )&\geq \vert a_{j_{t},j_{t}} \vert -R_{j_{t}}(A)+\omega _{j_{t}}+\frac{\det B_{j_{t}}}{\det\mu[A(\alpha)]} \\ &\geq \vert a_{j_{t},j_{t}} \vert -R_{j_{t}}(A)+\omega _{j_{t}}\geq \vert a_{j_{t},j_{t}} \vert -R_{j_{t}}(A)>0. \end{aligned} $$

Similarly,

21 $$ \begin{aligned}[b] \vert \widetilde{a}_{j_{t},j_{t}} \vert +R_{j_{t}}(A/\alpha )&\leq \vert a_{j_{t},j_{t}} \vert +R_{j_{t}}(A)-\omega _{j_{t}}-\frac{\det B_{j_{t}}}{\det\mu[A(\alpha)]} \\ &\leq \vert a_{j_{t},j_{t}} \vert +R_{j_{t}}(A)-\omega _{j_{t}}\leq \vert a_{j_{t},j_{t}} \vert -R_{j_{t}}(A). \end{aligned} $$

This completes the proof of case (i).

Next, we prove case (ii). Assume $A(\alpha)\notin H^{I}_{ \vert \alpha \vert }$, it then follows from Lemma 3 that $A(\alpha)$ has at least one diagonally equipotent principal submatrix. Let $A(\gamma)$ be the largest diagonally equipotent principal submatrix of the matrix $A(\alpha)$ for $\gamma=\alpha-\gamma'\subseteq\alpha$. Then $A(\gamma')$ has no diagonally equipotent principal submatrix and hence $A(\gamma')\in H^{I}_{ \vert \gamma' \vert }$ from Lemma 3. Since $A\in D_{n}$ and $A(\gamma)$ is the largest diagonally equipotent principal submatrix of the matrix $A(\alpha)$, it follows from Lemma 4 that ${A/\alpha}=A(\alpha'\cup\gamma ')/\gamma'$, where $\alpha'=\langle n\rangle-\alpha\subseteq\langle n\rangle$ and $A(\alpha^{\prime}\cup\gamma')$ is given in (10). Since $A(\gamma')\in H^{I}_{ \vert \gamma' \vert }\cap D_{ \vert \gamma ' \vert }$, it follows from the proof of case (i) that both (2) and (3) hold, which shows that the proof of case (ii) is completed. This completes the proof. □

### Theorem 4

*Given a matrix* $A=(a_{ij})\in D_{n}$ *and two sets* $\alpha=\{i_{1},i_{2},\ldots, i_{m}\}\subset\langle n\rangle$ *and* $\alpha '=\langle n\rangle-\alpha=\{j_{1},j_{2},\ldots, j_{l}\}\subset\langle n\rangle$ *with* $m+l=n$, *define* $\omega_{j_{t}}$ *as in* (1) *and* $A/\alpha=(\widetilde{a}_{j_{t},j_{s}})$, *if* $\omega_{j_{t}}>0$ *for all* $j_{t}\in\alpha'$, *then*

22 $$ \vert \widetilde{a}_{j_{t},j_{t}} \vert -R_{j_{t}}(A/\alpha )\geq \vert {a}_{j_{t},j_{t}} \vert -R_{j_{t}}(A)+\omega_{j_{t}}> \vert {a}_{j_{t},j_{t}} \vert -R_{j_{t}}(A)\geq0 $$

*and*

23 $$ \vert \widetilde{a}_{j_{t},j_{t}} \vert +R_{j_{t}}(A/\alpha )\leq \vert {a}_{j_{t},j_{t}} \vert +R_{j_{t}}(A)-\omega _{j_{t}}< \vert {a}_{j_{t},j_{t}} \vert +R_{j_{t}}(A). $$

### Proof

Using the same proof method as the one of Theorem 3, the conclusion of this theorem is obtained immediately. □

### Theorem 5

*Given a matrix* $A=(a_{ij})\in D_{n}$ *and two sets* $\alpha=\{i_{1},i_{2},\ldots, i_{m}\}\subset\langle n\rangle$ *and* $\alpha '=\langle n\rangle-\alpha=\{j_{1},j_{2},\ldots, j_{l}\}\subset\langle n\rangle$ *with* $m+l=n$, *define* $\omega_{j_{t}}$ *as in* (1), *if* $A(\alpha)$ *is nonsingular*, *then* $A/\alpha=(\widetilde {a}_{j_{t},j_{s}})$ *satisfies*

24 $$ \vert \widetilde{a}_{j_{t},j_{t}} \vert -R_{j_{t}}(A/\alpha )\geq \vert {a}_{j_{t},j_{t}} \vert -R_{j_{t}}(A)+\omega _{j_{t}}\geq \vert {a}_{j_{t},j_{t}} \vert -R_{j_{t}}(A)\geq0 $$

*and*

25 $$ \vert \widetilde{a}_{j_{t},j_{t}} \vert +R_{j_{t}}(A/\alpha )\leq \vert {a}_{j_{t},j_{t}} \vert +R_{j_{t}}(A)-\omega _{j_{t}}\leq \vert {a}_{j_{t},j_{t}} \vert +R_{j_{t}}(A). $$

### Proof

Similar to the proof of Theorem 3, one may derive the conclusion of this theorem. □

## The eigenvalue distribution of the Schur complement of nonstrictly diagonally dominant matrices

In this section, the result of Theorem 2 will be generalized to nonstrictly diagonally dominant matrices.

### Theorem 6

*Let* $A=(a_{ij})\in D_{n}$ *be nonsingular with real diagonal entries*, *and* $\alpha\subset\langle n\rangle$ *such that for all* $j\in\alpha '=\langle n\rangle-\alpha\subset\langle n\rangle$, $\vert {a}_{jj} \vert >R_{j}(A)=\sum_{k=1,k\neq j}^{n} \vert a_{jk} \vert $. *Then* $A/\alpha$ *and* $A(\alpha')$ *have the same number of eigenvalues whose real parts are greater* (*less*) *than* *w* (*resp*. −*w*), *where* *w* *is defined in* (4).

### Proof

Since $A\in D_{n}$ and is nonsingular, it follows from Lemma 2 that $A(\alpha)$ is nonsingular. Thus, $A/\alpha$ exists. Similar to the proof of Theorem 5 in [1], $\mu[A/\alpha]-wI$ is diagonally dominant coming from (2) in Theorem 1, so is $A/\alpha-wI$. Further, (2) indicates that $\widetilde{a}_{j_{t},j_{t}}-w>0$ if and only if $\widetilde {a}_{j_{t},j_{t}}>0$. This implies that $\vert J_{+}(A/\alpha) \vert = \vert J_{+}(A/\alpha-wI) \vert $. Thus, by the Gers̆gorin theorem, $A/\alpha-wI$ has $\vert J_{+}(A/\alpha ) \vert $ eigenvalues with positive real part (on the open right-half complex plane). On the other hand, the eigenvalues of $A/\alpha-wI$ are the eigenvalues of $A/\alpha$ minus *w*, so $A/\alpha$ has $\vert J_{+}(A/\alpha) \vert $ eigenvalues with positive real part greater than *w*. Again, since $[A(\alpha )]^{-1}=[A/\alpha]^{-1}$ (see, e.g., p.184 in [8]) and further that $A/\alpha$ and $[A/\alpha]^{-1}$ have the same number of eigenvalues with positive real part. It follows from Corollary 3 of [1] that $A/\alpha$ and $A(\alpha')$ have the same number of eigenvalues whose positive real parts are greater than *w*. For the number of negative parts of eigenvalues, the argument above still works with $-A/\alpha$ in place of $A/\alpha$. □

### Theorem 7

*Given a matrix* $A=(a_{ij})\in D_{n}$ *with real diagonal entries*, *and two sets* $\alpha\subset\langle n\rangle$ *and* $\alpha'=\langle n\rangle -\alpha\subset\langle n\rangle$, *if*

26 $$ \vartheta=\min_{i\in\alpha, j\in \alpha'} \biggl[ \frac{ \vert a_{ii} \vert -R_{i}(A)}{ \vert a_{ii} \vert }\sum_{k\in \alpha} \vert a_{jk} \vert \biggr]>0, $$

*then* $A/\alpha$ *and* $A(\alpha')$ *have the same number of eigenvalues whose real parts are greater* (*less*) *than* *ϑ* (*resp*. −*ϑ*).

### Proof

Since $A\in D_{n}$ and $\vartheta=\min_{i\in\alpha, j\in \alpha'} [\frac{ \vert a_{ii} \vert -R_{i}(A)}{ \vert a_{ii} \vert }\sum_{k\in \alpha} \vert a_{jk} \vert ]>0$, $\vert a_{ii} \vert >R_{i}(A)$ for all $i\in\alpha$ and $A(\alpha')$ has no diagonally equipotent principal submatrix. Then it follows from Lemma 2 that *A* is nonsingular, so is $A(\alpha)$. Thus, $A/\alpha$ exists. Similar to the proof of Theorem 6, the conclusion of this theorem is derived immediately. □

## Numerical examples

In this section, some numerical examples are given to demonstrate the effectiveness of the results obtained in this paper.

### Example 1

Consider the following $7\times7$ matrix:

27 $$ A=\left [ \textstyle\begin{array}{ccccccc} 1 & -1 & 0 & 0 & 0 & 0 & 0 \\ 1 & -2 & -1 & 0 & 0 & 0 & 0 \\ 0 & 1 & -2 & -1 & 0 & 0 & 0 \\ 0 & 0 & 1 & -3 & 1 & 0 & 0 \\ 0 & 0 & 0 & 1 & 3 & -1 & 0 \\ 0 & 0 & 0 & 0 & 1 & -3 & -1 \\ 0 & 0 & 0 & 0 & 0 & 1 & 2 \end{array}\displaystyle \right ]. $$

Let $\alpha=\{1,2,3\}$. Then $\alpha'=\{4,5,6,7\}$. It is verified that *A* and $A(\alpha)$ are both nonsingular and (13) holds. Thus, *A* satisfies the conditions of Theorems 3 and 5. According to Theorems 3 and 5, both (2) and (3) hold.

In what follows we will verify the conclusions of Theorems 3 and 5. Direct computations yield the following results:

28 $$ \begin{gathered} A/\alpha=\left [ \textstyle\begin{array}{cccc} -4 & 1 & 0 & 0\\ 1 & 3 & -1 & 0\\ 0 & 1 & -3 & -1 \\ 0 & 0 & 1 & 2 \end{array}\displaystyle \right ], \\ \omega_{4}= \omega_{5}=\omega_{6}= \omega_{7}=0, \\ \vert \widetilde{a}_{4,4} \vert -R_{4}(A/ \alpha)=3, \quad\quad \vert {a}_{4,4} \vert -R_{4}(A)=1, \\ \vert \widetilde{a}_{5,5} \vert -R_{5}(A/ \alpha)=1, \quad\quad \vert {a}_{5,5} \vert -R_{5}(A)=1, \\ \vert \widetilde{a}_{6,6} \vert -R_{6}(A/\alpha)=1,\quad\quad \vert {a}_{6,6} \vert -R_{6}(A)=1, \\ \vert \widetilde{a}_{7,7} \vert -R_{5}(A/ \alpha)=1, \quad\quad \vert {a}_{7,7} \vert -R_{7}(A)=1, \\ \vert \widetilde{a}_{4,4} \vert +R_{4}(A/ \alpha)=5, \quad\quad \vert {a}_{4,4} \vert +R_{4}(A)=5, \\ \vert \widetilde{a}_{5,5} \vert +R_{5}(A/ \alpha)=5, \quad\quad \vert {a}_{5,5} \vert +R_{5}(A)=5, \\ \vert \widetilde{a}_{6,6} \vert +R_{6}(A/ \alpha)=5, \quad\quad \vert {a}_{6,6} \vert +R_{6}(A)=5, \\ \vert \widetilde{a}_{7,7} \vert +R_{5}(A/ \alpha)=5, \quad\quad \vert {a}_{7,7} \vert +R_{7}(A)=5. \end{gathered} $$

Thus,

29 $$ \begin{gathered} \vert \widetilde{a}_{4,4} \vert -R_{4}(A/\alpha)\geq \vert {a}_{4,4} \vert -R_{4}(A)+\omega_{4}\geq \vert {a}_{4,4} \vert -R_{4}(A)\geq0, \\ \vert \widetilde{a}_{5,5} \vert -R_{5}(A/\alpha)\geq \vert {a}_{5,5} \vert -R_{5}(A)+\omega_{5} \geq \vert {a}_{5,5} \vert -R_{5}(A)\geq0, \\ \vert \widetilde{a}_{6,6} \vert -R_{6}(A/\alpha)\geq \vert {a}_{6,6} \vert -R_{6}(A)+\omega_{6} \geq \vert {a}_{6,6} \vert -R_{6}(A)\geq0, \\ \vert \widetilde{a}_{7,7} \vert -R_{5}(A/\alpha)\geq \vert {a}_{7,7} \vert -R_{7}(A)+\omega_{7} \geq \vert {a}_{7,7} \vert -R_{7}(A)\geq0 \end{gathered} $$

and

30 $$ \begin{gathered} \vert \widetilde{a}_{4,4} \vert +R_{4}(A/\alpha)\leq \vert {a}_{4,4} \vert +R_{4}(A)-\omega_{4}\leq \vert {a}_{4,4} \vert +R_{4}(A), \\ \vert \widetilde{a}_{5,5} \vert +R_{5}(A/\alpha)\leq \vert {a}_{5,5} \vert +R_{5}(A)-\omega_{5} \leq \vert {a}_{5,5} \vert +R_{5}(A), \\ \vert \widetilde{a}_{6,6} \vert +R_{6}(A/\alpha)\leq \vert {a}_{6,6} \vert +R_{6}(A)-\omega_{6} \leq \vert {a}_{6,6} \vert +R_{6}(A), \\ \vert \widetilde{a}_{7,7} \vert +R_{5}(A/\alpha)\leq \vert {a}_{7,7} \vert +R_{7}(A)-\omega_{7} \leq \vert {a}_{7,7} \vert +R_{7}(A). \end{gathered} $$

(29) and (30) show that the conclusions of Theorems 3 and 5 are true.

Let us investigate Theorem 6. Since $\alpha=\{1,2,3\}$ and $\alpha'=\{4,5,6,7\}$ such that *A* satisfies the condition of Theorem 6, $A/\alpha$ and $A(\alpha')$ have the same number of eigenvalues whose real parts are greater (less) than *w* (resp. −*w*).

Now, we verify the effectiveness of the conclusion by direct computations. We get $w=1$; further, we have that the eigenvalues of $A/\alpha$ and $A(\alpha')$ are −4.127, −2.626, 3.000, 1.753 and −3.106, −2.671, 3.023, 1.754, respectively. Thus, $A/\alpha$ and $A(\alpha')$ have two eigenvalues whose real parts are greater (less) than 1 (resp. −1). This shows that the conclusion of Theorem 6 is true.

### Example 2

Consider the following $6\times6$ matrix:

31 $$ A=\left [ \textstyle\begin{array}{cccccc} -5 & -1 & 0 & 1 & 0 & -1 \\ 1 & 5 & -1 & 1 & 1 & 0 \\ 1 & 1 & 5 & -1 & 0 & 1 \\ -2 & 1 & 1 & -6 & -1 & 1 \\ 1 & -2 & -1 & 1 & 6 & -1 \\ -1 & 1 & 1 & 1 & 1 & 5 \end{array}\displaystyle \right ]. $$

Assume $\alpha=\{1,2,3\}$. Then $\alpha'=\{4,5,6\}$. By direct computations, we get $\omega_{4}=\omega_{5}=0.80$ and $\omega_{6}=0.60$. Since $\omega_{j_{t}}>0$ for all $j_{t}\in\alpha'$, it follows from Theorem 6 that both (2) and (3) hold.

The following will show the effectiveness of Theorem 6 by direct computations. Since

32 $$ \begin{gathered} A/\alpha=\left [ \textstyle\begin{array}{ccc} -6.50 & -1.25 & 1.25\\ 1.47 & 6.41 & -1.06\\ 0.742 & 0.790 & 5.05 \end{array}\displaystyle \right ], \\ \vert \widetilde{a}_{4,4} \vert -R_{4}(A/ \alpha)=4, \quad\quad \vert {a}_{4,4} \vert -R_{4}(A)=0, \\ \vert \widetilde{a}_{5,5} \vert -R_{5}(A/ \alpha)=3.88,\quad\quad \vert {a}_{5,5} \vert -R_{5}(A)=0, \\ \vert \widetilde{a}_{6,6} \vert -R_{6}(A/\alpha)=3.52,\quad\quad \vert {a}_{6,6} \vert -R_{6}(A)=0, \\ \vert \widetilde{a}_{4,4} \vert +R_{4}(A/ \alpha)=9,\quad\quad \vert {a}_{4,4} \vert +R_{4}(A)=12, \\ \vert \widetilde{a}_{5,5} \vert +R_{5}(A/ \alpha)=8.94, \quad\quad \vert {a}_{5,5} \vert +R_{5}(A)=12, \\ \vert \widetilde{a}_{6,6} \vert +R_{6}(A/ \alpha)=6.58, \quad\quad \vert {a}_{6,6} \vert +R_{6}(A)=10. \end{gathered} $$

Thus,

33 $$\begin{aligned}& \vert \widetilde{a}_{4,4} \vert -R_{4}(A/\alpha)\geq \vert {a}_{4,4} \vert -R_{4}(A)+\omega_{4}\geq \vert {a}_{4,4} \vert -R_{4}(A)\geq0, \\& \vert \widetilde{a}_{5,5} \vert -R_{5}(A/\alpha)\geq \vert {a}_{5,5} \vert -R_{5}(A)+\omega_{5} \geq \vert {a}_{5,5} \vert -R_{5}(A)\geq0, \\& \vert \widetilde{a}_{6,6} \vert -R_{6}(A/\alpha)\geq \vert {a}_{6,6} \vert -R_{6}(A)+\omega_{6} \geq \vert {a}_{6,6} \vert -R_{6}(A)\geq0 \end{aligned}$$

and

34 $$\begin{aligned}& \vert \widetilde{a}_{4,4} \vert +R_{4}(A/\alpha)\leq \vert {a}_{4,4} \vert +R_{4}(A)-\omega_{4}\leq \vert {a}_{4,4} \vert +R_{4}(A), \\& \vert \widetilde{a}_{5,5} \vert +R_{5}(A/\alpha)\leq \vert {a}_{5,5} \vert +R_{5}(A)-\omega_{5} \leq \vert {a}_{5,5} \vert +R_{5}(A), \\& \vert \widetilde{a}_{6,6} \vert +R_{6}(A/ \alpha)\leq \vert {a}_{6,6} \vert +R_{6}(A)- \omega_{6}\leq \vert {a}_{6,6} \vert +R_{6}(A). \end{aligned}$$

(33) and (34) show that the conclusions of Theorem 6 are true.

It follows that we will verify the effectiveness of Theorem 7. Direct computations give $\vartheta=0.60>0$. According to Theorem 7, $A/\alpha$ and $A(\alpha')$ have the same number of eigenvalues whose real parts are greater (less) than *ϑ* (resp. −*ϑ*).

By direct computations, eigenvalues of $A/\alpha$ and $A(\alpha')$ are −6.422, $5.691 + 0.571i$, $5.691- 0.571i$ and −5.992, $5.496 + 0.815i$, $5.496-0.815i$, respectively. As a result, $A/\alpha$ and $A(\alpha')$ have two eigenvalues whose real parts are greater than 0.6 and have an eigenvalue whose real part is less than −0.6, respectively. This shows that the conclusion of Theorem 7 is true.

## Conclusions

This mainly studies the disc separation and the eigenvalue location of some special matrices. Firstly, the result on the Geršgorin disc separation from the origin for strictly diagonally dominant matrices and their Schur complements in [1] is extended to nonstrictly diagonally dominant matrices and their Schur complements to reveal that under some conditions the separation of the Schur complement of a nonstrictly diagonally dominant matrix is greater than that of the original grand matrix. Secondly, some significant conclusions are derived to establish the eigenvalue distribution of the Schur complements for nonstrictly diagonally dominant matrices. Finally, some examples are provided to demonstrate the effectiveness of some theoretical results obtained in this paper.
